# Burnout Profiles Among Young Researchers: A Latent Profile Analysis

**DOI:** 10.3389/fpsyg.2022.839728

**Published:** 2022-05-27

**Authors:** Anke Boone, Tinne Vander Elst, Sofie Vandenbroeck, Lode Godderis

**Affiliations:** ^1^Department of Public Health and Primary Care, Centre for Environment and Health, University of Leuven (KU Leuven), Leuven, Belgium; ^2^Knowledge, Information and Research Center, IDEWE Group (External Service for Prevention and Protection at Work), Leuven, Belgium; ^3^Department of Social Psychology, Tilburg University, Tilburg, Netherlands

**Keywords:** burnout—professional, mental health, PhD students, Job Demands—Resources model, researchers, job resources and demands

## Abstract

**Introduction:**

Burnout is a growing problem among young researchers, affecting individuals, organizations and society. Our study aims to identify burnout profiles and highlight the corresponding job demands and resources, resulting in recommendations to reduce burnout risk in the academic context.

**Methods:**

This cross-sectional study collected data from young researchers (*n* = 1,123) at five Flemish universities through an online survey measuring burnout risk, work engagement, sleeping behavior, and the most prominent job demands (e.g., publication pressure) and resources (e.g., social support). We conducted Latent Profile Analysis (LPA) to identify burnout profiles in young researchers and subsequently compared these groups on job demands and resources patterns.

**Results:**

Five burnout profiles were identified: (1) High Burnout Risk (9.3%), (2) Cynical (30.1%), (3) Overextended (2.3%), (4) Low Burnout Risk (34.8%), and (5) No Burnout Risk (23.6%). Each burnout profile was associated with a different pattern of job demands and resources. For instance, high levels of meaningfulness (OR = −1.96) decreased the odds to being classified in the Cynical profile.

**Conclusion:**

Our findings show that the Cynical profile corresponds to a relatively high number of young researchers, which may imply that they are particularly vulnerable to the cynicism dimension of burnout. Additionally, work-life interference and perceived publication pressure seemed the most significant predictors of burnout risk, while meaningfulness, social support from supervisor and learning opportunities played an important protective role.

## Introduction

Stress-related diseases are a growing concern in the academic work environment, affecting young researchers in particular (Watts and Robertson, 2011; Levecque et al., 2017; Woolston, 2017; Evans et al., 2018; Mattijssen et al., 2020a,b; Tikkanen et al., 2021). Although some stress is beneficial for personal and professional growth (Pappa et al., 2020), prolonged exposure to stress may lead to mental health problems such as burnout, a condition characterized by extreme exhaustion, cynicism and reduced professional efficacy (Maslach et al., 2001). According to several studies, 30%–40% of researchers experience mental health problems (Divaris et al., 2012; Gloria and Steinhardt, 2016; Levecque et al., 2017; Evans et al., 2018; Mattijssen et al., 2020b), which indicates a higher risk compared to the general population (Levecque et al., 2017; Barry et al., 2018; Nagy et al., 2019). High workload (Barkhuizen et al., 2014; Mattijssen et al., 2020a), competitive work environments (Levecque et al., 2017), job insecurity (Petersen et al., 2012; Nagy et al., 2019; Guidetti et al., 2021) and high publication pressure (Miller et al., 2011; Tijdink et al., 2013) are some of the factors leading to high stress levels among this occupational group.

These difficult working conditions further deteriorated during the COVID-19 pandemic (Byrom, 2020; Gibson et al., 2020; Woolston, 2020; Gewin, 2021). Multiple studies have shown that researchers reported higher stress levels, anxiety and burnout risk during this crisis (Byrom, 2020; Levine and Rathmell, 2020; Sharma et al., 2020; Gewin, 2021). Some reasons for this were that experiments and fieldtrips were canceled or postponed, data collection was restricted due to travel limitations and study designs had to be modified (Fleming et al., 2020; Sharma et al., 2020; Woolston, 2020; Gewin, 2021). This situation is reflected in the fact that approximately 80% of postdoctoral researchers believe the pandemic seriously hindered their research (Woolston, 2020).

In addition to a negative impact on their research work, mental health problems and burnout among researchers affect both researchers and society. First, it has a negative impact on the individual level: their personal life (e.g., relationship and children; Kusurkar et al., 2020), overall health (Levecque et al., 2017), and future career development (Barthauer et al., 2020). Furthermore, stress and high burnout risk (i.e., emotional exhaustion in particular) were found to correlate positively with sleep problems and insomnia (Kusurkar et al., 2020). Second, there are consequences on the organizational and societal level. Mental health issues are positively correlated with lower work engagement (Taris et al., 2017; Tikkanen et al., 2021), reduced productivity, more turnover intentions and financial costs (Dorenkamp and Weiß, 2018; McAlpine et al., 2018; Nagy et al., 2019; Barthauer et al., 2020; Cho and Hayter, 2021; Jaksztat et al., 2021; Tikkanen et al., 2021).

In recent years, there has been an increase in burnout research following a person-centered approach, such as Latent Profile Analysis (LPA; Sandrin et al., 2021; Van der Vaart and de Beer, 2021; Kalamara and Richardson, 2022). LPA is a categorical latent variable modeling approach that identifies latent subpopulations based on certain variables (Spurk et al., 2020). It is particularly beneficial when it comes to facilitating research findings into practice and designing interventions (Leiter and Maslach, 2016; Mäkikangas and Kinnunen, 2016). More specifically, LPA implies that respondents are clustered into several burnout profiles based on their responses to a series of burnout risk indicators (Leiter and Maslach, 2016; Spurk et al., 2020).

For instance, Leiter and Maslach (2016) argue that this approach identifies qualitative differences between respondents, which may differentiate between people who score high on emotional exhaustion from people who score high on cynicism or reduced professional efficacy. The main added value of this is that it allows researchers to examine whether these profiles differ in terms of both patterns of burnout dimensions and patterns of job demands (e.g., workload) and job resources (e.g., social support; Leiter and Maslach, 2016).

Despite an increase in burnout studies in the academic work context and the surge in a person-centered approach to investigating burnout risk, little is still known about young researchers’ latent burnout profiles (Mäkikangas and Kinnunen, 2016; Spurk et al., 2020). To our knowledge, only one study has conducted LPA among young researchers (Tikkanen et al., 2021). However, it did not include an analysis on how the identified profiles differed in terms of job demands and resources. Based on former burnout studies among young researchers, we expect that the LPA including young researchers’ specific challenges (e.g., publication pressure, job insecurity; Tijdink et al., 2013; Guidetti et al., 2021) will provide us with new information that is vital to design, develop and implement tailored burnout interventions. Due to this groups’ specific challenges, the results of our LPA are expected to be different from recent studies that have conducted LPA among other occupational groups (i.e., teachers, fire workers; Sandrin et al., 2021; Kalamara and Richardson, 2022).

Furthermore, most former burnout studies in the academic context focused on academic staff in general (Kinman, 2008; Barkhuizen et al., 2014), included only PhD researchers (Devine and Hunter, 2017; Levecque et al., 2017; Nagy et al., 2019) or integrated only one discipline or university (Waaijer et al., 2016; Nagy et al., 2019). Additionally, the group of postdoctoral researchers (Gloria and Steinhardt, 2016; Guthrie et al., 2017; Vekkaila et al., 2018; Guidetti et al., 2021) and the Flemish context (Levecque et al., 2017) have been poorly addressed in existing burnout literature. Hence, this is the first study that uses LPA to examine burnout profiles and their relation to the most prominent job demands and resources for young researchers in Flanders, encompassing a large sample of doctoral and postdoctoral researchers from different disciplines at all (five) Flemish universities.

Given the high personal, financial and societal importance of researchers’ well-being, it is important to address young researchers’ high burnout risk (Nagy et al., 2019). Overall, our study aims to (1) identify the most prevalent burnout profiles among young researchers (i.e., PhD and postdoctoral researchers), (2) investigate the corresponding job demands and resources for each burnout profile, and (3) provide recommendations for structural interventions based on the burnout profiles that can support young researchers and reduce their burnout risk. Additionally, our results will contribute to the growing body of evidence that investigates burnout risk using LPA and elaborates on the use of this methodology (Leiter and Maslach, 2016; Spurk et al., 2020).

## Background and Theoretical Framework

### Young Researchers in Flanders, Belgium

To remain competitive, Flanders has implemented a range of incentives to support and stimulate young researchers, such as an increase in number of doctoral mandates, the establishment of doctoral schools and the introduction of the “dehousse” scholarship, which is exempted from taxes (Groenvynck, 2009; Levecque et al., 2017; ECOOM, 2021; Luwel, 2021). This resulted in a continuous and substantial increase in young researchers, from 6.321 PhD students and 1.512 postdoctoral researchers in 2004 to 9.733 PhD students and 3.791 postdoctoral researchers in 2020 (ECOOM, 2021). With regard to the gender balance, women represented 48% of PhD students in 2020, compared to 45% in 2004 (ECOOM, 2021). Unfortunately, the share of women decreases with seniority level as in 2020 women comprised only 39% of postdoctoral researchers, even though this percentage had increased from 34% in 2004 (ECOOM, 2021).

Flanders has five universities, which are all research institutes and they receive most of their funding from the Flemish government (Levecque et al., 2017; Luwel, 2021). Similar to many European countries, the majority of young researchers in Flanders has a formal full-time employment contract and a full scholarship, providing them with more secure incomes compared to United States systems (Coimbra Group, 2016; Levecque et al., 2017). In addition, in Flanders compared to the United States system, the registration fees are lower, the focus is more on “on-the-job” training and the duration of a doctoral program is shorter (i.e., 4–5 years compared to 7 years; Coimbra Group, 2016; Levecque et al., 2017; Barry et al., 2018).

### Burnout: Concept, Measurement, and Interpretation

The most often used definition of burnout was introduced by Maslach and Jackson (1981) and defines burnout risk by three dimensions: emotional exhaustion, cynicism and reduced professional efficacy (Maslach and Jackson, 1981; Maslach et al., 2001). Emotional exhaustion refers to a chronic state of physical and emotional depletion, also described as feelings of extreme fatigue. Cynicism or depersonalization is the experience of feeling detached and developing negative feelings toward one’s job. Reduced professional efficacy means developing a negative image about one’s own professional competence (Maslach and Jackson, 1981; Maslach et al., 2001). These three burnout dimensions constitute the Maslach Burnout Inventory (MBI), which is the “golden standard” to measure burnout risk (Maslach and Jackson, 1981). Nevertheless, the way results from the MBI should be treated and interpreted has been subject to discussion (Dyrbye et al., 2009).

In their recent study, Leiter and Maslach (2016) recommend a person-centered approach using LPA, in which burnout risk is treated as a complex and multidimensional phenomenon where emotional exhaustion, cynicism and reduced professional efficacy manifest themselves differently in each individual (Dyrbye et al., 2009). According to the authors, LPA can specifically contribute to translating MBI results into recommendations and interventions, which can prevent and reduce the risk of burnout among high-risk occupational groups (i.e., young researchers) by clustering participants into several burnout profiles based on their responses to a series of burnout indicators (Leiter and Maslach, 2016).

In their study, Leiter and Maslach (2016) identified five burnout profiles among healthcare workers: Burned-out (high on exhaustion, cynicism and reduced professional efficacy), Overextended (high on exhaustion only), Disengaged (high on cynicism only), Ineffective (high on reduced professional efficacy only), and Engagement (low on exhaustion, cynicism and reduced professional efficacy; Leiter and Maslach, 2016). Additionally, each burnout profile was associated with various patterns of organizational factors. For example, the Overextended profile showed a stronger association with high workload, while the Disengaged profile showed a stronger association with low job resources such as social support, influence at work and meaningfulness (Leiter and Maslach, 2016).

Tikkanen et al. (2021) investigated engagement and burnout risk among medical doctoral students through LPA. Four distinct profiles were identified: high engagement–low burnout, high engagement–moderate burnout, moderate engagement–moderate burnout, and moderate engagement–high burnout (Tikkanen et al., 2021). Additionally, some other studies conducted LPA among university students (Lee et al., 2017; Salmela-Aro and Read, 2017; Portoghese et al., 2018). Portoghese et al. (2018) identified three burnout profiles among Italian students (Burned-out, Overextended, and Engaged) and Salmela-Aro and Read (2017) found four profiles among Finish students (Burned-out, Engaged-Exhausted, Ineffective, and Engaged). Finally, Lee et al. (2017) conducted LPA for Korean university students and they identified three profiles (High burnout risk, Middle burnout risk, and Low burnout risk).

### Job Demands-Resources Model

Occupational health research provides us with the holistic and integrated Job Demands-Resources (JD-R) model on burnout risk and work engagement (Demerouti et al., 2001; Bakker and Demerouti, 2007). Work engagement is defined as “a positive, fulfilling, work-related state of mind that is characterized by vigor, dedication, and absorption” (Schaufeli et al., 2002, p. 74). The idea behind the JD-R model is that every occupation has its own specific job demands and job resources that contribute to burnout risk and/or work engagement (Demerouti et al., 2001). While job demands refer to “those physical, social, or organizational aspects of the job that require sustained physical or mental effort and are associated with certain physiological and psychological costs” (Demerouti et al., 2001, p. 501), job resources may “(1) be functional in achieving work goals, (2) reduce job demands at the associated physiological and psychological costs, (3) stimulate personal growth and development” (Demerouti et al., 2001, p. 501).

The JD-R model has been widely used to gain insights into the most important job demands and resources in specific occupational groups, in relation to burnout risk (Bakker and Demerouti, 2007, 2008; Bakker et al., 2014). Research shows that job resources are important predictors of work engagement, while both job demands and job resources affect burnout risk (Demerouti et al., 2001; Schaufeli and Bakker, 2004). Work engagement and burnout risk relate negatively with each other, and also job demands and job resources have a negative correlation (Schaufeli and Bakker, 2004; Bezuidenhout and Cilliers, 2010; Barkhuizen et al., 2014). In addition, only burnout risk, not work engagement, appears to be associated with sleeping problems (Schaufeli and Bakker, 2004; Ribeiro et al., 2018).

#### Main Job Demands for Young Researchers

Our study has selected the following most cited job demands in the academic work environment for young researchers based on former research: a high workload, work-life interference, continuous publication pressure and job insecurity.

##### Workload

Quantitative job demands, workload and hours worked are the most often cited job demands among researchers (Boyd et al., 2011; Barkhuizen et al., 2014; Waaijer et al., 2016; Mattijssen et al., 2020a; Cho and Hayter, 2021). In recent years, the workload has continuously increased implying unrealistic deadlines, constant time pressure and a rise in additional tasks (e.g., teaching, administrative duties, supervising; Waaijer et al., 2016; Cho and Hayter, 2021). Emotional exhaustion in particular seems to be strongly associated with high workload (Hunter and Devine, 2016).

##### Work-Life Interference

Not being able to maintain a healthy balance between work and personal life is another important job demand for young researchers (Fox et al., 2011; Bell et al., 2012; Kusurkar et al., 2020; Cho and Hayter, 2021; Jackman and Sisson, 2021). A *Nature* survey in 2019 revealed that 76% of respondents worked more than 41 h per week, leaving almost no time for family, friends or relaxation (Woolston, 2019). Rather than this being a personal decision, 46% of the respondents confirmed that the university culture promotes long hours and regular night work (Woolston, 2019).

##### Publication Pressure

The pressure to publish has evolved into the well-known “publish or perish” culture among researchers (Miller et al., 2011; Petersen et al., 2012; Waaijer et al., 2016). For young researchers, scientific publications are the performance standard, quantifying their academic achievements (Petersen et al., 2012). This emphasis on quantity, impact factors and citation numbers intensifies the competition between young researchers and results in high perceived publication pressure (Miller et al., 2011). Furthermore, significant associations were found between burnout risk and perceived publication pressure, with young researchers experiencing more publication pressure than their senior counterparts (Tijdink et al., 2013).

##### Job Insecurity

Young researchers often lack stable contracts and face challenges in obtaining funding (Boyd et al., 2011; Petersen et al., 2012; Reevy and Deason, 2014; Acker and Haque, 2015; Barry et al., 2018; Nagy et al., 2019), which contributes to the competitive environment and more occupational stress (Petersen et al., 2012; Denecke et al., 2016; Cho and Hayter, 2021). Besides finances, the insecurity also encompasses uncertain future career possibilities inside or outside academia (Acker and Haque, 2015; Waaijer et al., 2016; Woolston, 2017; Hayter and Parker, 2019; Nagy et al., 2019; Cho and Hayter, 2021). In recent years, there seems to be a trend toward an increase in temporary contracts, resulting in even more job insecurity (Waaijer et al., 2016). Furthermore, job insecurity in the academic sector seems to impact the cynicism dimension of burnout risk in particular (Guidetti et al., 2021).

#### Main Job Resources for Young Researchers

Based on previous research, our study identified the following most common job resources in the academic work environment: influence at work, learning opportunities, meaning of work and social support from colleagues and supervisor.

##### Influence at Work

Influence at work or job control is an umbrella concept encompassing perceived job autonomy, work control (i.e., time) and decision authority (Boyd et al., 2011; Park et al., 2014; Kusurkar et al., 2020). When researchers are not involved in the decision-making process or have no say in the content or organization of their work, this may lead to feelings of worthlessness and insignificance (Bell et al., 2012; Kusurkar et al., 2020; Chacón-Cuberos et al., 2021). On the other hand, when they have a sense of autonomy, they feel more engaged and enjoy doing their work (Jackman and Sisson, 2021).

##### Opportunities for Learning

Researchers enjoy to learn, take initiative, experiment and reflect with others over new ideas (Pyhältö et al., 2009). The learning environment can act as a source of inspiration and may give young researchers the opportunity to learn new skills and develop themselves professionally (Stubb et al., 2011).

##### Meaning of Work

Meaningfulness of work is related to how researchers perceive their work and why they do it (Stubb et al., 2012; Barry et al., 2018). Young researchers usually consider their work meaningful when they believe it leads to the enhancement of science and has societal impact (Stubb et al., 2011).

##### Social Support From Colleagues

Having colleagues that trust and support each other, have mutual respect, and value each other’s work has been reported to be highly important (May et al., 2004; Caesens et al., 2014; Barry et al., 2018; Vekkaila et al., 2018; Cho and Hayter, 2021). In addition, receiving positive feedback from peers or other academics also contributes to an increased self-esteem (Pyhältö et al., 2017; Jackman and Sisson, 2021).

##### Social Support From Supervisor

A supportive, responsive and accessible supervisor who stimulates, challenges and supports young researchers has been found to be crucial for their well-being, especially in the hierarchical academic work environment (Waaijer et al., 2016; Levecque et al., 2017; Pyhältö et al., 2017; Woolston, 2017; Cho and Hayter, 2021; Jackman and Sisson, 2021). Moreover, social support from the supervisor seems to reduce emotional exhaustion (Devine and Hunter, 2017) and can be an important buffer when job insecurity is high (Guidetti et al., 2021). On the other hand, a lack of social support from the supervisor shows a strong association with increased levels of cynicism (Vekkaila et al., 2018).

**Figure 1:**
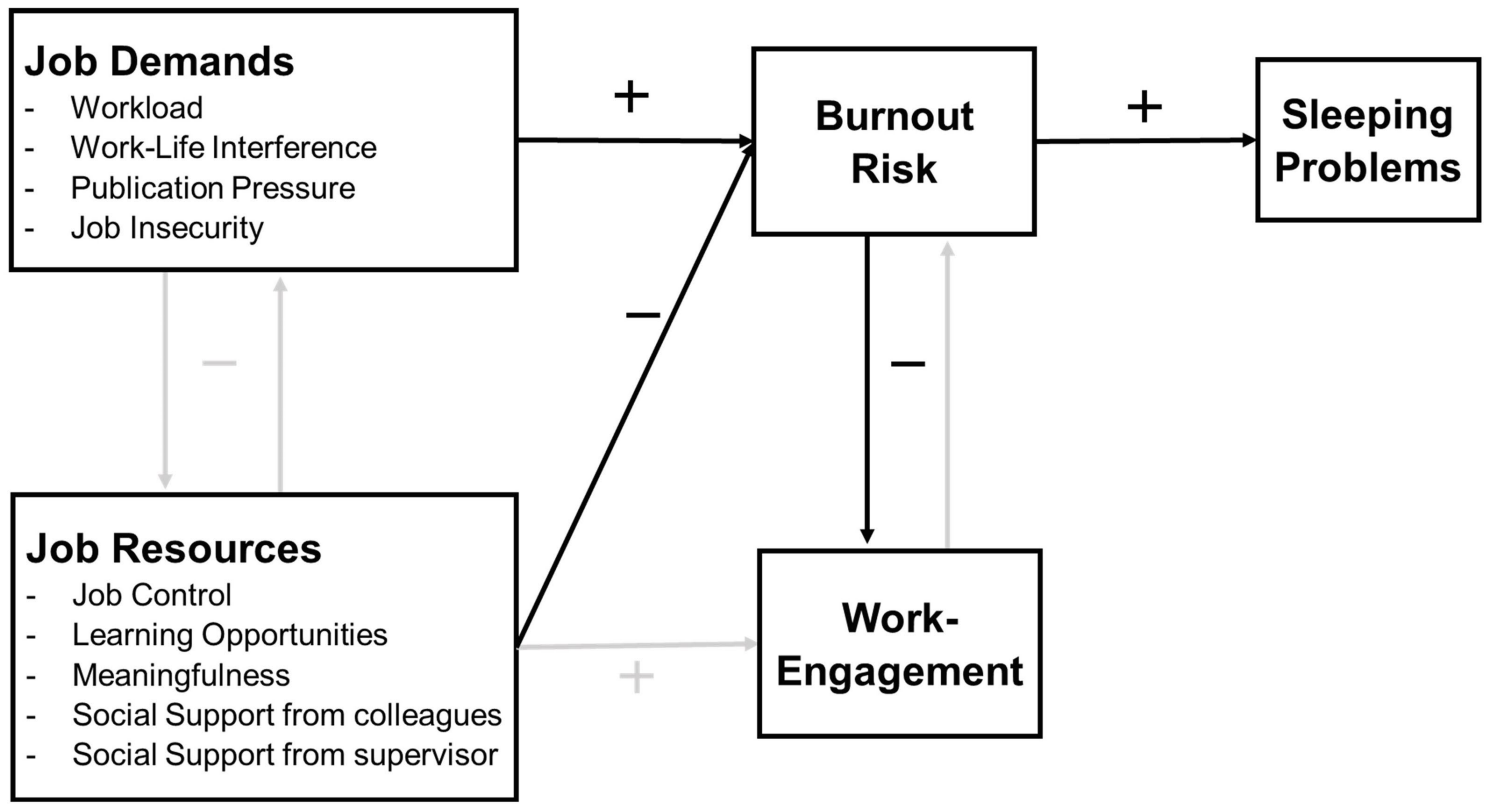
The adapted JD-R model (Schaufeli and Bakker, 2004; Lindblom et al., 2006). This study will focus on the black arrows, the arrows in gray will not be addressed.

Figure 1 provides a schematic overview of the JD-R model adapted to the academic context.

### Hypotheses

Based on previous research by Leiter and Maslach (2016), we expect that scores on the burnout dimensions will cluster into multiple latent burnout profiles, representing burnout risk as a multidimensional concept. We hypothesize that the first latent profile will score high on all burnout dimensions, labeled as the High Burnout Risk profile (*Hypothesis 1a*). The second profile will be characterized by high scores on cynicism and low scores on emotional exhaustion and professional efficacy, and will be labeled Cynical (*Hypothesis 1b*). The third profile will score high on emotional exhaustion and low on the two other burnout dimensions, and will be labeled Overextended (*Hypothesis 1c*). The fourth, Ineffective profile, will show high scores on the reduced professional efficacy dimension (*Hypothesis 1d*; Leiter and Maslach, 2016). The fifth, No Burnout Risk profile, will score low on all three burnout dimensions (*Hypothesis 1e*), which differs from Leiter and Maslach (2016), who labeled it as Engagement (Leiter and Maslach, 2016). We followed the recommendations of Schaufeli and Bakker (2004), who consider burnout risk and work engagement as two independent constructs, rather than Leiter and Maslach (2016), who view burnout risk and work engagement as two endpoints of the burnout risk continuum.

In addition, Leiter and Maslach (2016) give direction regarding differences in patterns of job demands and resources. Respondents that experience high job demands (i.e., workload, work-life interference, publication pressure and job insecurity) and low job resources (i.e., influence at work, learning opportunities, meaning of work, social support from colleagues and support from supervisor) will more likely belong to the High Burnout Risk profile (*Hypothesis 2a*; Leiter and Maslach, 2016). With regard to the three intermediate profiles, we expect that respondents scoring lower on job resources, influence at work, learning opportunities, meaning of work and social support are more likely to be categorized in the Cynical profile compared to the Overextended and Ineffective profile (*Hypothesis 2b*; Leiter and Maslach, 2016). In addition, we predict that a high score on workload will increase the likelihood to be categorized in the Overextended profile (*Hypothesis 2c*; Consiglio et al., 2014; Leiter and Maslach, 2016). Furthermore, the expectations for the Ineffective profile are less clear based on previous research, although it is likely to reflect a more negative set of scores compared to the No Burnout Risk profile (*Hypothesis 2d*; Leiter and Maslach, 2016).

Finally, we will investigate whether the five burnout profiles differ with respect to the outcomes of work engagement (Schaufeli and Bakker, 2004; Leiter and Maslach, 2016) and sleeping problems (Armon et al., 2008; Vela-Bueno et al., 2008). First, we expect that the High Burnout Risk profile will score the lowest on work engagement, followed by the Cynical profile. It is also hypothesized the No Burnout Risk profile will have the highest score, followed by the Ineffective and Overextended profile (*Hypothesis 3*; Leiter and Maslach, 2016). Second, we expect that respondents with high scores on sleeping problems will more likely belong to the High Burnout Risk profile, followed by the Overextended profile (Armon et al., 2008; Vela-Bueno et al., 2008; Kusurkar et al., 2020). Respondents in the No Burnout Risk, Cynical and Ineffective profiles are predicted to have the lowest scores on sleeping problems (*Hypothesis 4*; Armon et al., 2008; Vela-Bueno et al., 2008; Kusurkar et al., 2020).

## Methodology

### Procedure

All five Flemish universities participated in a cross-sectional survey study: University of Leuven (KU Leuven), Vrije Universiteit Brussel (VUB), Universiteit Hasselt (UHasselt), Universiteit Gent (UGent) and Universiteit Antwerpen (UAntwerpen). We applied a convenience sampling strategy, in which we contacted the universities’ doctoral schools and asked them to distribute our web-based survey to their PhD students and postdoctoral researchers from October to December 2020. Respondents could access the survey through a link in the doctoral school’s newsletter or an invitation mail. The survey was administered in English using the software package Qualtrics. We only included researchers who agreed with the informed consent, were working on a PhD or a postdoctoral research project, and were affiliated with one of the five universities. We added a quality check question (*This is a quality check question, please indicate ‘never’*) halfway throughout the survey to exclude respondents who did not carefully read the questions (Kung et al., 2018). Respondents were briefed about the study’s objectives by an informed consent, and participation was always anonymous and voluntary. Ethical approval was obtained from the Social and Societal Ethics Committee of the KU Leuven (G-2020-2388).

### Participants

In response to our online survey, we received a total of 1,465 answers, of which 1,123 met the inclusion and exclusion criteria. The final sample comprised 933 PhD students and 190 postdoctoral researchers, representing a response rate of approximately 10 and 5%, respectively (ECOOM, 2021). The percentage of missing data in this sample was 3%. According to Schafer (1999), a missing rate of 5% or less is insignificant (Schafer, 1999). Table 1 shows that the mean age was 29.17 years (*SD* = 5.49), 77% were in a relationship and 14% had children. Furthermore, 69% of our respondents were female. This number is higher compared to the proportion of women in our target population, which is 45% (ECOOM, 2021).

**Table 1:** Background information.

Characteristic	Item	*N*	%
**Socio-demographics**			
Gender	Female	765	69
	Male	348	31
Age	20–30 years	799	72
	31–40 years	270	24
	> 41 years	42	4
In a relationship	Yes	862	77
Children	Yes	156	14
**Work situation**			
Position	PhD student	933	83
	Postdoctoral researcher	190	17
Faculty	Medicine, Life Sciences or Health Studies	257	23
	Veterinary Medicine, Pharmaceutical, Biomedical or Biosciences	80	7
	Architecture, Arts and Philosophy	108	10
	Social, Communication and Political Sciences	112	10
	Law and Criminology	70	6
	Physical Education, Physiotherapy or Rehabilitation sciences	42	4
	Psychology or Educational Sciences	87	8
	Sciences (biology, statistics, mathematics)	155	14
	Engineering: technology, applied or bioscience	105	10
	Theology or Religious studies	9	1
	Business, Economics and Transportation	67	6
	Geography, Environmental Studies	3	0.5
	Other	3	0.5

### Instruments

#### Burnout Risk

“Burnout Risk” was measured using the 16-item *Maslach Burnout Inventory – General Survey (MBI-GS)*, created by Maslach and Jackson (1981). The MBI-GS is the most widely used burnout risk measurement instrument, assessing burnout risk by its three core dimensions: emotional exhaustion (five items), cynicism (five items) and reduced professional efficacy (six items; Urbina-Garcia, 2020). A five-point Likert scale was used to measure the frequency in which respondents experience feelings related to each scale, ranging from “never” (1) to “every day” (5; Maslach et al., 2018). We used a five-point Likert scale, instead of the original seven-point Likert scale, to ensure conformity throughout the scales used in our survey. The emotional exhaustion scale had an internal consistency (Cronbach’s alpha) of 0.89, the cynicism scale of 0.82 and the reduced professional efficacy scale of 0.79.

#### Predictors

Regarding the job demands, “workload” (four items) and “work-life interference” (five items) were measured using the corresponding scales of the *Copenhagen Psychosocial Questionnaire* (*COPSOQ*; Burr et al., 2019). The items were measured on a 5-point Likert scale ranging from “never” (1) to “always” (5). The “workload” scale had a Cronbach’s alpha of 0.77 and the “work-life balance” scale of 0.86. “Publication pressure” was examined using the stress scale (6 items) of the *Publication Pressure Questionnaire* (*PPQ*; Haven et al., 2019). This scale measures researcher’s perceived publication pressure. The items were measured on a 5-point Likert scale, ranging from “strongly disagree” (1) to “strongly agree” (Cronbach’s alpha = 0.77; 5). “Job insecurity” was measured using four items from the *Job Insecurity Scale* (*JIS*). This instrument assesses whether respondents believe their job is at risk (Vander Elst et al., 2014). The items were measured on a 5-point Likert scale, from “strongly disagree” (1) to “strongly agree” (Cronbach’s alpha = 0.91; 5).

Regarding the job resources, “influence at work” (six items), “learning opportunities” (three items), “meaning of work” (two items), “social support from colleagues” (five items) and “social support from supervisor” (three items) were assessed using the corresponding scales of the *COPSOQ* (Burr et al., 2019). The items were measured on a 5-point Likert scale from “never” (1) to “always” (5), with an exception for “meaning of work” whose items were scored on a 5-point Likert scale ranging from “strongly disagree” (1) to “strongly agree” (5). The Cronbach’s alphas were high for all scales: “influence at work” (0.77), “learning opportunities” (0.82), “meaning of work” (0.86), “social support from colleagues” (0.77) and “social support from supervisor” (0.84).

#### Outcomes

“Work engagement” (three items) was assessed using the *COPSOQ* (Burr et al., 2019). The items were measures on a 5-point Likert scale, ranging from “never” (1) to “every day” (5; Cronbach’s alpha = 0.72). “Sleeping problems” (four items) were assessed using the corresponding scale of the *COPSOQ* (Burr et al., 2019). All items were measured on a 5-point Likert scale, ranging from “never” (1) to “every night”(5; Cronbach’s alpha = 0.80).

#### Control Variables

We controlled for age, gender (1 = female, 0 = male/other) and job position (1 = PhD student, 0 = Postdoctoral researcher) in the analyses, because previous research has indicated that being younger may be related to a higher burnout risk, and that woman may score higher on emotional exhaustion, while men score higher on cynicism (Maslach et al., 2001; Lindblom et al., 2006; Watts and Robertson, 2011).

A series of Confirmatory Factor Analyses with robust maximum likelihood estimation (using MPlus 8.5; Muthén and Muthén, 2017b) verified the expected dimensionality of the study variables. Given the large number of scales, we conducted separated analyses for (1) the dimensions of burnout risk and the outcomes (with factors emotional exhaustion, cynicism, reduced professional efficacy, work engagement and sleeping problems, *χ*^2^(220) = 1683.51, *p* < 0.001, CFI = 0.87, NNFI = 0.85, RMSEA = 0.08, SRMR = 0.06) and (2) the job demands and resources (with factors workload, work-life interference, publication pressure, job insecurity, influence at work, learning opportunities, meaning of work, social support from colleagues and social support from supervisor, χ^2^(629) = 1917.91, *p* < 0.001, CFI = 0.93, NNFI = 0.92, RMSEA = 0.04, SRMR = 0.05). For each group of scales, the hypothesized measurement model fit the data better than several alternative measurement models (see Table 2). However, we acknowledge that the fit indices provided mixed evidence for the fit of the measurement model for burnout and the outcomes. While the SRMR of 0.06 indicated an excellent fit (values up to 0.09) and the RMSEA of 0.08 indicated a good fit (values up to 0.08), the CFI and NNFI did not reach the cut-off for adequate fit of 0.90. Hence, we inspected for sources of misfit based on the modification indices provided by MPlus. They showed that the fit could be particularly improved by allowing the error terms of two pairs of items of the cynicism scale (item 1 and 2; item 3 and 4) to covary. Adding these covariances can also be explained in terms of content as they refer to the same aspect of the cynicism scale (enthusiasm and contribution respectively; see also Schutte et al., 2000). These specifications resulted in a final measurement model for burnout risk and the outcomes with an adequate fit [*χ*^2^(218) = 1167.351, *p* < 0.001, CFI = 0.92, NNFI = 0.90, RMSEA = 0.06, SRMR = 0.06].

**Table 2:** Results of the confirmatory factor analyses.

Measurement model	Latent factors	*χ* ^2^	CFI	NNFI	RMSEA	SRMR	Comparison of nested models	Satorra-Bentler scaled Δ*χ*^2^
*Burnout risk and outcomes*
1a. Hypothesized 5-factor model	Emotional exhaustion, cynicism, reduced professional efficacy, work engagement, sleeping problems	*χ*^2^(220) = 1683.51^***^	0.87	0.85	0.08	0.06	–	–
1b. Hypothesized 5-factor model—respecified	Model 1a in which the error terms of items 1 and 2, and of items 4 and 5 of the Cynicism scale were allowed to covary	*χ*^2^(218) = 1167.35^***^	0.92	0.90	0.06	0.06	–	–
2. Alternative 3-factor model	Burnout risk, work engagement, sleeping problems	*χ*^2^(228) = 4125.58^***^	0.66	0.63	0.12	0.14	Model 2 vs. model 1a	Δχ2(8) = 2233.74^***^
3. Alternative 2-factor model	Burnout risk, work engagement, sleeping problems	*χ*^2^(229) = 3756.94^***^	0.69	0.66	0.12	0.09	Model 3 vs. model 1a	Δχ2(9) = 1869.07^***^
4. Alternative 1-factor model	General factor	*χ*^2^(230) = 4841.70^***^	0.60	0.56	0.13	0.11	Model 4 vs. model 1a	Δχ2(10) = 2588.84^***^
*Job demands and resources*
1a. Hypothesized 9-factor model	Workload, work-life interference, publication pressure, job insecurity, influence at work, learning opportunities, meaning of work, social support from colleagues and social support from supervisor	*χ*^2^(629) = 1917.91^***^	0.93	0.92	0.04	0.05	–	–
2. Alternative 2-factor model	Job demands, job resources	*χ*^2^(664) = 9644.23^***^	0.48	0.45	0.11	0.10	Model 2 vs. model 1a	Δχ2(35) = 6040.79^***^
3. Alternative 1-factor model	General factor	*χ*^2^(665) = 11408.51^***^	0.37	0.34	0.12	0.12	Model 3 vs. model 1a	Δχ2(36) = 6809.35^***^

***p < 0.001.

### Data Analysis

First, we tested the assumption of normality by examining the distribution of each observed variable for skewness (index greater than 3) and kurtosis (index higher than 10). We checked for multicollinearity by screening for bivariate correlations higher than 0.85 (Weston and Gore, 2006) and by examining the Variance Inflation Index (VIF; Alin, 2010). The analyses, performed with SPSS v27 confirmed that there was no evidence for problems with normality or multicollinearity. Descriptive analyses were performed with basic SPSS functions. We calculated mean scores and standard deviations and conducted correlational analyses on all study variables.

We conducted LPA in MPlus 8.5 (Muthén and Muthén, 2017a) to identify latent profiles of respondents with a similar pattern on the three burnout dimensions, and subsequently compared these groups with respect to the hypothesized predictors and distal outcomes. We followed the three-step procedure presented by Asparouhov and Muthén (2014). In the first step, regular Latent Cluster Analysis was conducted, in which the mean scores of emotional exhaustion, cynicism and reduced professional efficacy were entered as the manifest indicators of the latent classes. This is a similar approach to Leiter and Maslach (2016), thus further increasing the comparability of our results. All LPAs were conducted using the maximum likelihood estimator with robust standard errors (MLR), and we relied on the Full Information Maximum Likelihood (FIML) method to deal with missing values in the data. We increased the number of random start values in the first and second step of the optimization to 500 and 50, respectively, while the number of iterations in the first step of the optimization was set at 50 (Geiser, 2012). We tested and compared a series of LPA models, starting with a model with one profile and adding a profile at each step until no better model was found. Each model was checked for its quality, model fit and the interpretability of its profiles to select the best profile solution (Geiser, 2012). In terms of quality, we checked the entropy (should be as close to 1 as possible). Relative model fit, reflecting an LPA model’s fit compared to a nested model with one class less, was assessed based on the Akaike Information Criterion (AIC), the Bayesian Information Criterion (BIC), the sample size-adjusted BIC (SSA-BIC), the Vuong-Lo–Mendell–Rubin likelihood ratio test (VLMR) and the Bootstrap likelihood ratio test (BLRT). In the second step, respondents were assigned to profiles based on their most likely class membership using the latent class posterior distribution (Asparouhov and Muthén, 2014).

In the third step, we investigated relationships of the discrete burnout profiles with the auxiliary variables, either acting as predictors or distal outcomes, while taking into account the misclassification of individuals in the final latent profiles. To test the predictors, the R3STEP command in MPlus was used (Asparouhov and Muthén, 2014). This command conducts multinomial logistic regressions assessing whether an increase in the predictor is related to a higher probability of belonging to a certain burnout profile over another profile. To model the distal outcomes, the DU3STEP command in MPlus was used (Asparouhov and Muthén, 2014). This command determines whether the means of the outcome variables differ across the latent burnout profiles. The predictors and distal outcomes were tested separately (Lanza et al., 2013).

## Results

### Descriptive Results

Supplementary Table A.1 (available as Appendix) displays the means, standard deviations, reliabilities (Cronbach’s alpha in parentheses) and Pearson’s correlations for all study variables.

### Latent Profile Analysis

First, we ran a set of LPA models with an increasing number of latent profiles and compared their fit based on multiple fit indices (see Table 3). Based on BIC and BLRT, the model with 4 latent profiles showed the best fit: BIC was lowest for the four-profile model, and the BLRT demonstrated a better fit for the four-profile model compared to the model with three profiles, whereas the model with five profiles could not further increase model fit. However, the five-profile solution fit data best based on the VLMRT, demonstrated by the significant values up to a solution with five profiles. Further, SSA-BIC values were lowest for the four- and five-profile solution with a minor advantage for the four-profile model. Moreover, AIC values decreased with increasing numbers of profiles. Next, entropy was largest for the model with three profiles, decreased for the four-profile model and subsequently increased again for the five-profile model.

**Table 3:** Comparing model fit for different burnout profiles.

Model	LL	*df*	AIC	BIC	SSA-BIC	VLMRT	BLRT	VLMRT (*p*)	BLRT (*p*)	Entropy
1 profile	−3732.821	6	7477.642	7507.785	7488.727	–	–	<0.001	<0.001	–
2 profiles	−3332.114	10	6684.229	6734.466	6702.704	801.413	801.413	<0.001	<0.001	0.750
3 profiles	−3261.204	14	6550.408	6620.741	6576.273	141.820	141.820	<0.001	<0.001	0.710
4 profiles	−3240.419	18	6516.838	6607.265	6550.092	41.571	41.571	0.008	<0.001	0.661
5 profiles	−3233.65	22	6511.301	6621.823	6551.945	13.537	13.537	0.012	0.054	0.682
6 profiles	−3228.552	26	6509.103	6639.721	6557.138	10.197	10.197	0.233	0.176	0.671
7 profiles	−3220.840	30	6501.680	6652.393	6557.104	15.423	15.423	0.483	0.014	0.685

*LL, log-likelihood; df, degrees of freedom; AIC, Akaike Information Criterion; BIC, Bayesian Information Criterion; SSA-BIC, sample size-adjusted BIC; VLMR, Vuong-Lo–Mendell–Rubin likelihood ratio test; and BLRT, bootstrapped log-likelihood ratio test. Lower AIC and (SSA-)BIC values indicate better fitting models. Significant values of *p* for the VLMRT and BLRT indicate that the model is a worse fit compared to a model with 1 fewer class. Entropy is a measure of how accurate a model is at classifying people into latent profiles*.

**Figure 2:**
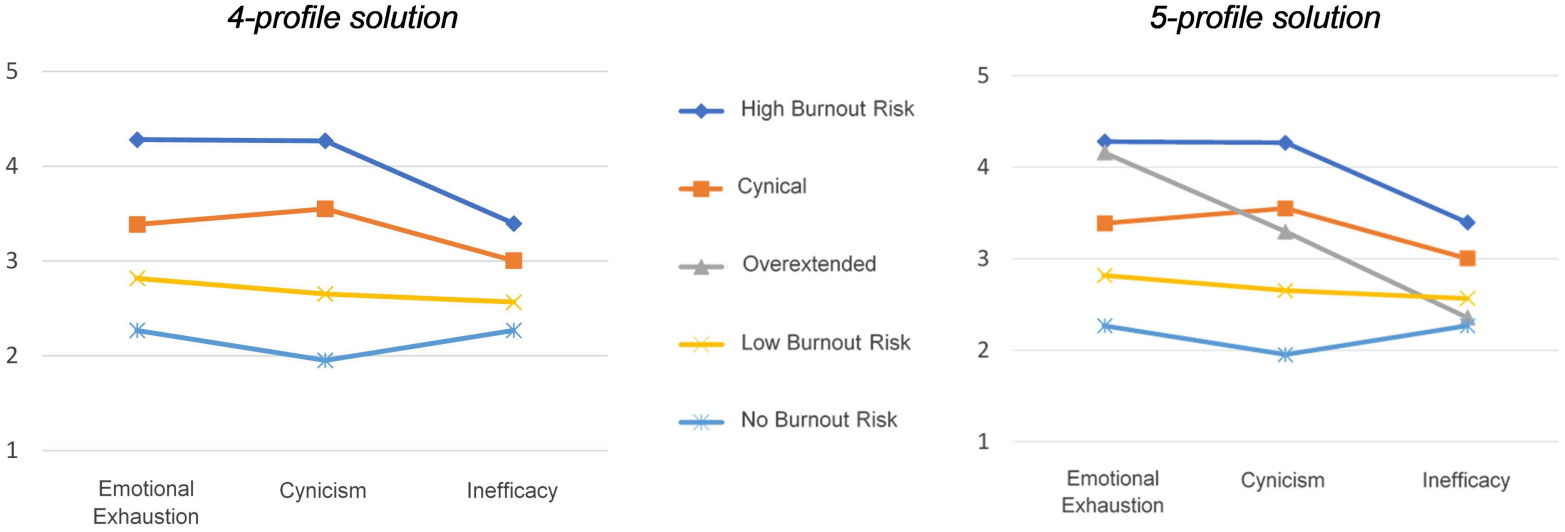
Latent profiles of burnout: 4-profile solution and 5-profile solution.

Based on model fit and entropy, we believed a choice had to be made between the four-profile and the five-profile solution. Hence, we further inspected the interpretability of the profiles. A graphical display of the estimated means for the four and five latent profiles solution of burnout risk can be found in Figure 2. While one of the profiles in the five-profile solution consisted of only 2.3% of the sample (but still larger than 1%, the cut-off presented by Bennett et al., 2016), this profile was not redundant to the other profiles (see Table 3; Bennett et al., 2016). Moreover, the profiles in the five-profile solution corresponded closely with the five profiles previously identified by Leiter and Maslach (2016) (33). Hence, we decided to select the five-profile model as the final model (see Figure 2).

In the selected 5-profile solution, as predicted, the first latent profile of burnout risk was labeled as “High Burnout Risk” (*Hypothesis 1a*) and contained 9.3% of the respondents (*n* = 104). The averages for emotional exhaustion and cynicism were 4.28 and 4.26, respectively, meaning that these respondents experienced emotional exhaustion and cynicism between (4) *often* and (5) *every day*. Their average score of reduced professional efficacy was 3.40, laying between the response categories (3) *sometimes* and (4) *often*. The second profile was labeled as “Cynical,” because of the slightly higher score on cynicism compared to the scores for the other dimensions of burnout, which was in line with *Hypothesis 1b*. This profile consisted of 30.1% of the respondents (*n* = 338). The third profile was characterized by a relatively high average score on emotional exhaustion, as predicted in *Hypothesis 1c*, on average experiencing cynicism (3) *sometimes* and a relatively low score on reduced professional efficacy. Only 2.3% of the respondents (*n* = 26) were categorized in this “Overextended” profile. The fourth profile was the largest latent profile, consisting of 34.8% of the respondents (*n* = 391). This profile was typified by average scores on the three burnout dimensions between (2) *rarely* and (3) *sometimes*, and was therefore labeled as “Low Burnout Risk.” This only partly supported *Hypothesis 1d*, as we expected this profile to score high on reduced professional efficacy only. Finally, the fifth profile—labeled as “No Burnout Risk”—contained 23.6% of the respondents (*n* = 265). Members of this profile on average experienced emotional exhaustion, cynicism and reduced professional efficacy only *rarely*. This was in line with *Hypothesis 1e*.

**Figure 3:**
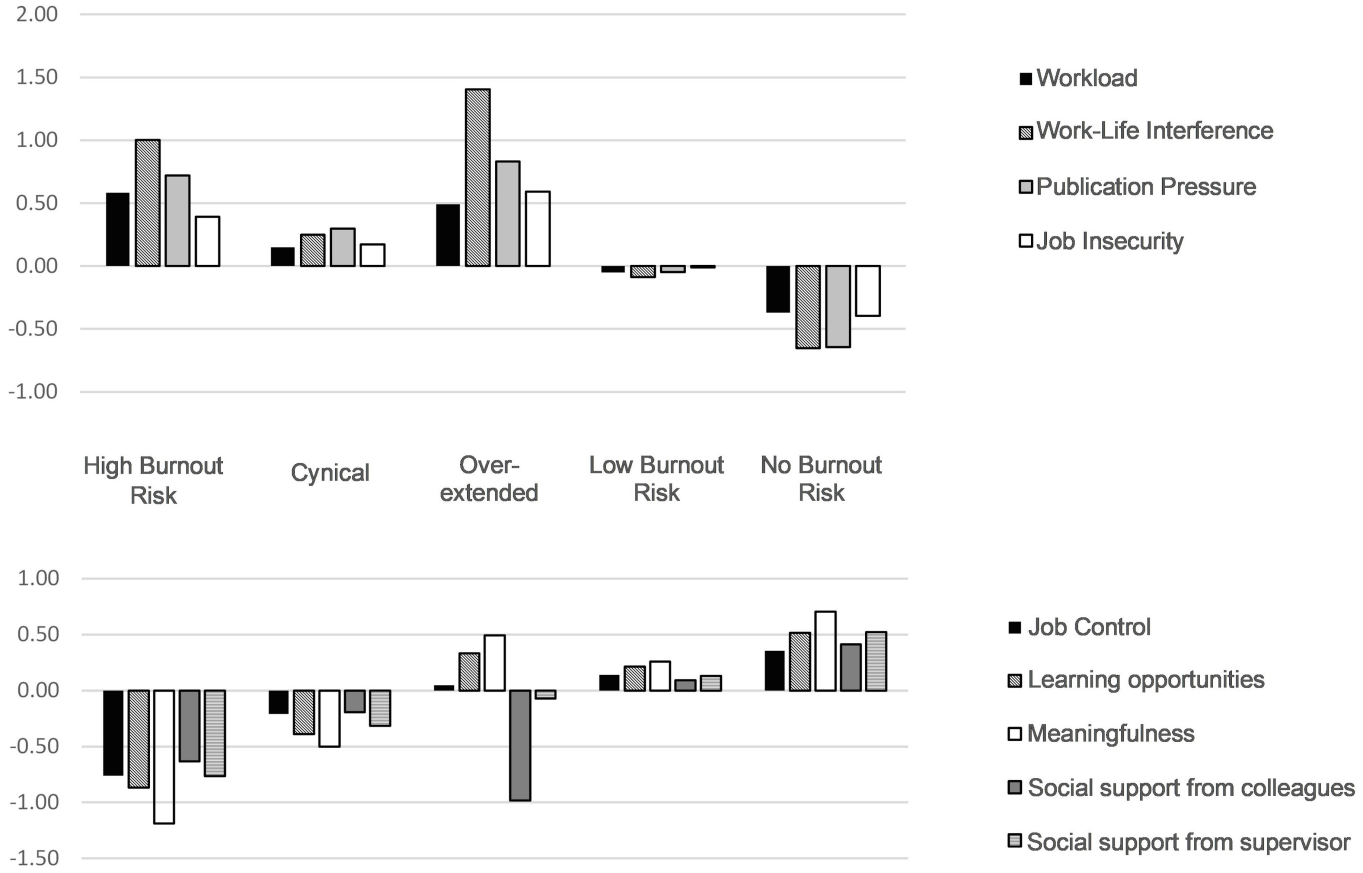
Standardized means of the predictors by latent burnout profile.

Next, we examined whether the job demands, job resources and control variables had a significant association with the burnout profiles using a multinomial logistic regression analysis (See Table 4). The profile “No Burnout Risk” was used as the reference category. Table 4 displays the odds ratios and the logistic regression coefficients for all predictors of latent burnout profile membership. To aid interpretation, Figure 3 presents the differences in standardized means of the predictors by burnout profile. As predicted in *Hypothesis 2a*, young researchers experiencing more work-life interference (*OR* = 75.65), publication pressure (*OR* = 9.11) and job insecurity (*OR* = 3.56) were more likely to be categorized in the High Burnout Risk profile compared to the No Burnout Risk profile, after controlling for the effects of age, gender and position. Unexpectedly, however, workload did not predict membership in the High Burnout Risk group. Concerning the job resources, researchers that scored lower for learning opportunities (*OR* = 0.15), meaning of work (*OR* = 0.001), social support from colleagues (*OR* = 0.36) and social support from the supervisor (*OR* = 0.34) were more likely to belong to the High Burnout Risk profile in comparison with the No Burnout Risk profile. This was also in line with *Hypothesis 2a*. However, unexpectedly, influence at work was not associated with the High Burnout Risk profile.

**Table 4:** Logistic regression coefficients for latent class analysis with covariates with the No Burnout Risk profile as the reference group (R3STEP).

Predictor	High burnout risk				Cynical				Overextended				Low burnout risk			
	Coeff.	*SE*	*p*	*OR*	Coeff.	*SE*	*p*	*OR*	Coeff.	*SE*	*p*	*OR*	Coeff.	*SE*	*p*	*OR*
Age	−0.174	0.110	0.114	0.840	−0.149	0.063	0.018	0.862	0.239	0.072	0.001	1.270	−0.017	0.044	0.695	0.983
Gender (female)	−0.027	0.688	0.969	0.974	−0.358	0.530	0.499	0.699	6.834	1.585	<0.001	928.965	−0.222	0.355	0.532	0.801
Position (PhD)	3.614	1.208	0.003	37.125	2.521	0.843	0.003	12.443	−3.434	1.868	0.066	0.032	1.407	0.572	0.014	4.082
Workload	0.532	0.554	0.336	1.703	0.104	0.526	0.844	1.109	0.786	0.959	0.413	2.194	−0.109	0.327	0.739	0.897
Work-life interference	4.326	0.660	<0.001	75.652	2.610	0.521	<0.001	13.596	7.736	1.485	<0.001	2288.509	1.263	0.288	<0.001	3.536
Publication pressure	2.210	0.691	0.001	9.112	1.824	0.474	<0.001	6.196	0.099	0.643	0.877	1.104	1.184	0.372	0.001	3.268
Job insecurity	1.269	0.325	<0.001	3.559	0.906	0.267	0.001	2.475	1.412	0.589	0.017	4.102	0.606	0.210	0.004	1.833
Influence at work	−0.227	0.587	0.700	0.797	0.101	0.501	0.840	1.106	−3.821	1.226	0.002	0.022	0.218	0.370	0.554	1.244
Learning opportunities	−1.922	0.560	0.001	0.146	−1.521	0.440	0.001	0.218	−1.528	0.847	0.071	0.217	−0.263	0.306	0.391	0.769
Meaningfulness	−6.526	0.724	<0.001	0.001	−5.191	0.692	<0.001	0.006	−1.958	0.998	0.050	0.141	−2.433	0.490	<0.001	0.088
Social support from colleagues	−1.011	0.472	0.032	0.364	−0.616	0.400	0.123	0.540	−4.258	1.298	0.001	0.014	−0.489	0.331	0.140	0.613
Social support from supervisor	−1.067	0.380	0.005	0.344	−0.933	0.271	0.001	0.393	1.932	0.454	<0.001	6.900	−0.478	0.205	0.020	0.620

Furthermore, our results partly supported *Hypothesis 2b*, in which we predicted that experiencing low levels of influence at work, meaning of work, social support from colleagues and social support from the supervisor would increase the odds to be clustered within the Cynical profile, compared to the two other intermediate burnout profiles. For instance, researchers scoring high on meaning of work (*OR* = −1.96) and social support from the supervisor (*OR* = −4.26) were significantly less likely to belong to the Cynical profile. Surprisingly though, this was not the case for influence at work and social support from colleagues. In addition, *Hypothesis 2c* was not confirmed, as workload was not significantly related to membership of the Overextended profile. Moreover, regarding *Hypothesis 2d*, members of Low Burnout Risk profile reflected a more negative set of scores compared to the No Burnout Risk profile. Consequently, this hypothesis was confirmed, although we did not label it as an Ineffective profile.

Finally, we investigated whether the five burnout profiles differed with respect to sleeping problems and work engagement. The results of the equality tests of the means across the latent profiles using the DU3STEP command in MPlus (see Table 5) show that the mean scores of both sleeping problems (*χ*^2^ = 203.71, *p* < 0.001) and work engagement (*χ*^2^ = 748.49, *p* < 0.001) differed significantly across the five burnout profiles. With respect to work engagement, our results generally supported *Hypothesis 3*, as the High Burnout Risk profile scored lowest, followed by the Cynical profile. However, we expected more difference between the Cynical and the Overextended profiles, which however did not differ from each other. The No Burnout Risk profile scored the highest on work engagement, followed by the Low Burnout Risk profile. Regarding sleeping problems, we predicted in *Hypothesis 4* that the High Burnout Risk profile would score highest, followed by the Overextended profile. However, our results show that the Overextended profile scored highest, followed by the High Burnout Risk profile. The No Burnout Risk profile scored the lowest on sleeping problems, followed by the Low Burnout Risk profile and the Cynical profile.

**Table 5:** Results of the latent profile analysis with distal outcomes: outcome means and comparisons between burnout profiles (DU3STEP).

Distal outcome	High burnout risk (profile 1)		Cynical (profile 2)		Overextended (profile 3)		Low burnout risk (profile 4)		No burnout risk (profile 5)		Overall *χ*^2^	Differences between profiles
	Mean	*SE*	Mean	*SE*	Mean	*SE*	Mean	*SE*	Mean	*SE*		
Sleeping problems	3.321	0.142	2.825	0.065	3.944	0.136	2.811	0.061	2.180	0.061	*χ*^2^ = 203.7, *p* < 0.001	3 > 1 > 2 = 4 > 5
Work engagement	2.358	0.068	2.947	0.032	2.975	0.224	3.553	0.030	3.870	0.034	*χ*^2^ = 748.49, *p* < 0.001	1 < 2 = 3 < 4 < 5

## Discussion

### The Identified Burnout Profiles Among Young Researchers

LPA generated five burnout profiles among young researchers: (1) High Burnout Risk (i.e., high on all three dimensions; *Hypothesis 1a*), (2) Cynical (i.e., high on cynicism in particular; *Hypothesis 1b*), (3) Overextended (i.e., high on emotional exhaustion in particular; *Hypothesis 1c*), (4) Low Burnout Risk (i.e., relatively low on all three dimensions; *Hypothesis 1d*), (5) No Burnout Risk (i.e., very low on all three dimensions; *Hypothesis 1e*). These results support previous research that conceptualizes burnout as a complex and multidimensional phenomenon using LPA, in which the three burnout dimensions manifest themselves differently in each individual (Leiter and Maslach, 2016; Mäkikangas and Kinnunen, 2016; Lee et al., 2017; Salmela-Aro and Read, 2017; Portoghese et al., 2018; Sandrin et al., 2021; Van der Vaart and de Beer, 2021; Kalamara and Richardson, 2022).

A major distinction from Leiter and Maslach (2016) is that our findings did not include an Ineffective profile (i.e., high on reduced professional efficacy only). Instead, we found a Low Burnout Risk profile, showing relatively low scores on all three burnout dimensions (*Hypothesis 1d*). This might mean that reduced professional efficacy is not the main problem faced by young researchers in our sample experiencing high levels of chronic stress. This finding is in agreement with Portoghese et al. (2018), who investigated burnout risk among Italian students, and similarly did not find an Ineffective profile. Because these authors investigated a sample (i.e., university students) more similar to ours than the sample of Leiter and Maslach (2016) (i.e., healthcare workers), we consider it a possibility that young researchers might be less susceptible to this type of experiences (Portoghese et al., 2018).

Additionally, the Overextended and the Cynical profile showed similar characteristics with the ones identified by Leiter and Maslach (2016). This implies that one subgroup of our sample feels exhausted (while maintaining low levels of cynicism and reduced professional efficacy), whereas another group feels highly cynical about their work (despite low levels of exhaustion and reduced professional efficacy). However, and in contrast with former research (Leiter and Maslach, 2016; Portoghese et al., 2018; Kalamara and Richardson, 2022), our study reported about three times more people in the Cynical profile and three times fewer in the Overextended profile. The observed difference may indicate that our sample is at a greater risk of becoming cynical than exhausted, compared to other occupational groups (e.g., healthcare workers, university students or teachers; Leiter and Maslach, 2016; Portoghese et al., 2018; Kalamara and Richardson, 2022). In this case, interventions that target the cynicism dimension of burnout should be prioritized.

Furthermore, our findings show that the Overextended group consists of a significantly higher number of women, which corroborates some previous studies, where women score slightly higher on emotional exhaustion (Maslach et al., 2001; Lindblom et al., 2006; Watts and Robertson, 2011; Mattijssen et al., 2020b). Yet, the evidence is mixed, as in some studies male postgraduates are more likely to experience high levels of exhaustion (Tikkanen et al., 2021), while others show no association between gender and exhaustion (Hunter and Devine, 2016). Nonetheless, caution with the interpretation of the results of the Overextended profile is required as it only consisted of 2.3% of the sample. This is, however, still larger than the 1% cut-off presented by Bennett et al. (2016).

### Relation With Organizational Constructs

As expected, our findings showed that high scores on work-life interference, publication pressure and job insecurity, and low scores on learning opportunities, meaningfulness and social support from colleagues and supervisor were strong predictors of belonging to the High Burnout Risk profile (*Hypothesis 2a*). This gives further support to the Job Demands-Resources model that high job demands and low job resources increase risk of burnout (Demerouti et al., 2001; Schaufeli and Bakker, 2004; Bakker and Demerouti, 2007; Leiter and Maslach, 2016; Salmela-Aro and Read, 2017).

Our research has also shown that each burnout profile was associated with a different pattern of organizational factors (i.e., job demands and job resources), which corroborates the findings of former LPA studies (Leiter and Maslach, 2016; Mäkikangas and Kinnunen, 2016; Salmela-Aro and Read, 2017). With regard to the three intermediate profiles, the Cynical profile showed the strongest association with meaningfulness, followed by learning opportunities and social support from the supervisor (*Hypothesis 2b*). One unexpected finding was that low social support from colleagues did not predict membership to the Cynical profile. This could be attributed to the fact that while support from colleagues is important, it is not sufficient to protect against membership in the Cynical profile. This can especially be true in the academic setting, with strong hierarchical relations and the reliance of young researchers on their supervisor. For example, a recent study indicated that cynicism was high among postdoctoral researchers who mentioned tensions in their supervisory relationships (Vekkaila et al., 2018). Consequently, training and improving the supervising skills of supervisors deserves to be a priority.

Surprisingly, however, workload did not predict membership in the High Burnout Risk or the Overextended profile (*Hypothesis 2c*), which seems to contrast with previous research indicating a significant impact of workload on burnout risk (Boyd et al., 2011; Barkhuizen et al., 2014; Leiter and Maslach, 2016; Waaijer et al., 2016; Mattijssen et al., 2020b; Cho and Hayter, 2021). We believe that this can be explained by the inclusion of work-life interference and publication pressure in this LPA, as we controlled for these two job demands, both of which showed high correlation with workload. In addition, and in accordance with our expectations, members of the Low Burnout Risk profile reflected a more negative set of scores compared to the No Burnout Risk profile (*Hypothesis 2d*).

Another surprising finding is that job control did not significantly protect against membership in the High Burnout Risk and Cynical profiles, which differs from Leiter and Maslach (2016). We argue that because young researchers inherently have a high degree of job autonomy, job resources such as meaningfulness, learning opportunities and social support may be more effective to address the risk of burnout for this occupational group, compared to an increased emphasis on influence at work or job autonomy.

Finally, work-life interference and perceived publication pressure stand out as important predictors of membership in the High Burnout Risk profile, which is consistent with previous studies (Fox et al., 2011; Miller et al., 2011; Bell et al., 2012; Petersen et al., 2012; Tijdink et al., 2013; Kusurkar et al., 2020; Cho and Hayter, 2021; Jackman and Sisson, 2021). Moreover, our results show that these two job demands are more significant predictors of the High Burnout Risk and the Cynical profile compared to workload. This finding creates opportunities to focus on interventions targeting work-life interference and publication pressure when addressing burnout risk among this occupational group. It has to be acknowledged, however, that the covid-19 pandemic might have exaggerated the impact of work-life interference as many researchers were obliged to work from home, further blurring the lines between personal and professional lives (Gibson et al., 2020).

### Relationship With Work Engagement and Sleeping Problems

Our findings have categorized two profiles as the endpoints of the burnout risk continuum, with one scoring high on all burnout dimensions (i.e., High Burnout Risk profile) and the other scoring low on all dimensions (i.e., No Burnout Risk profile). This is consistent with previous studies (Leiter and Maslach, 2016; Mäkikangas and Kinnunen, 2016; Salmela-Aro and Read, 2017; Portoghese et al., 2018; Kusurkar et al., 2020; Tikkanen et al., 2021; Kalamara and Richardson, 2022). However, our categorization differs from Leiter and Maslach (2016), who labeled the endpoints as the Burnout and the Engaged profile. This difference in labeling can be attributed to the use of a different conceptual framework of burnout risk. Leiter and Maslach (2016) based their study on a continuum between burnout risk and work engagement, implying these two constructs are dependent on one scale. Instead, we followed the recommendations of Schaufeli and Bakker (2004), who considered burnout risk and work engagement as two independent constructs. This implies that burnout risk and work engagement exhibit different patterns of causes, for which different intervention strategies should be used. Tikkanen et al. (2021) have also followed this latter approach, but they included study engagement in their LPA to identify the burnout profiles, while our study only included work-engagement as an outcome. Nevertheless, our findings show that burnout risk and work engagement negatively relate to each other. The High Burnout Risk profile followed by both the Cynical and Overextended profiles scored the lowest on work engagement, and the No Burnout Risk profile scored the highest (*Hypothesis 3*). With regard to sleeping problems, the Overextended profile scored the highest, followed by the High Burnout Risk profile (*Hypothesis 4*). These results match those of other studies (Armon et al., 2008; Vela-Bueno et al., 2008; Kusurkar et al., 2020), which have indicated a positive association between sleeping problems and high burnout risk.

### Recommendations

Our findings urge governments and institutions to prioritise the mental health of young researchers, as many experience an increased or high risk of burnout. Based on our findings, we recommend to follow a systems approach with regard to the design, development and implementation of burnout interventions, which includes burnout prevention as well as treatment.

A highly recommended strategy for prevention is to raise awareness, remove the stigma and expand knowledge among all actors involved (Levecque et al., 2017; Mattijssen et al., 2020b). Not only should policy makers and supervisors increase their knowledge but also young researchers should be educated about how to take care of their mental well-being, how to detect the early signs of burnout and how to seek help (Salmela-Aro and Read, 2017; Mattijssen et al., 2020b). The PhD curricula should incorporate courses on mental health, occupational stress and coping strategies, and this should start as early as the first year of the PhD process (Salmela-Aro and Read, 2017).

Furthermore, we recommend investing in creating meaning, providing learning opportunities and enhancing social support to prevent high levels of cynicism. As low social support from the supervisor seems an important predictor of burnout risk, supervisors should be trained to supervise constructively, recognize mental health issues and mitigate when problems arise (Cornér et al., 2017; Mattijssen et al., 2020b). In addition, a recent study has shown that social support from the supervisor can be an important buffer when job insecurity is high (Guidetti et al., 2021). It should be noted that supervisors themselves might be experiencing high stress levels, hence, structures should be in place to also provide them with the requested support (Mattijssen et al., 2020b). Furthermore, professional coaching sessions, easy-to-reach ombudspersons or peer mentoring programs are frequently suggested as a way to increase social support (Gardner, 2007; Stubb et al., 2012; Cornér et al., 2017; Barry et al., 2018).

Our findings also suggest that interventions targeting work-home interference and publication pressure have the potential to mitigate burnout risk among young researchers. For example, providing childcare on campus is one approach to facilitate a healthy work-life balance (Gibson et al., 2020), or promoting regular working hours rather than working overtime. With regard to publication pressure, we recommend to re-evaluate publication expectations (Gibson et al., 2020), and to provide structural support for improving writing skills and to make the writing process collaborative rather than individualist (i.e., peer review group sessions; Barry et al., 2018).

With regard to treatment, governments and institutions could take measures to protect young researchers who have experienced burnout or who are recovering from burnout. This may include a more systematic data collection on burnout numbers (Levecque et al., 2017) and setting up support systems, such as burnout treatment options (Levecque et al., 2017), or appointing psychologists, who are specialized in the circumstances and experiences of young researchers (Mattijssen et al., 2020b).

### Study Contributions

Given that human capital is the most valuable resource for countries with a “knowledge economy,” monitoring and addressing the mental health of young researchers should be a priority. Our study contributed to burnout research in the academic context in three ways. First, we followed a person-centered approach to burnout, using LPA, which provided insights into the multidimensionality of burnout (i.e., multiple burnout profiles) and facilitated the translation from theory into practical recommendations. Second, our findings documented the associations between the identified profiles and its main predictors (i.e., job demands and resources). The inclusion of these predictors is important, as it provides information on specific organizational factors that should be addressed in new interventions. Third, our study contributed to the growing body of evidence investing burnout using LPA and elaborated on the use of this methodology.

## Limitations

We should note several limitations of our research and how we addressed them. First, our study had a cross-sectional design, which limits the conclusions that can be drawn. A longitudinal study could have revealed changes over time, eliminating cohort effects and providing more information on the evolution of burnout dimensions within each identified burnout profile. Second, the incorporated validated questionnaires (i.e., MBI, COPSOQ, TIS, and JIS) measure respondent’s perceptions of their own behavior through self-rapportage, which is prone to social desirability bias. Third, we should mention that none of our analyses diagnose burnout. Although the profiles are identified in relation to each other, they do not fulfill clinical criteria. Hence, we can only discuss “burnout risk” and not “burnout.” Fourth, we standardized all Likert scales to five-point Likert scales to make completion of the survey more consistent for respondents, which also meant that we adjusted the MBI’s seven-point Likert scale. To overcome potential bias due to this, we conducted an additional factor analysis, which revealed no problems with validity. Fifth, the Latent Profile Analysis assumed the error terms of the indicators to be uncorrelated (“local interdependence”). However, when testing this assumption using both class-specific and class-invariant associations between the error terms of the indicators, we quickly encountered convergence issues due to the computational intensity of these tests (Asparouhov and Muthén, 2015). Future research using very large samples may overcome this issue. Sixth, respondents participated voluntary in the study, implying the possibility of selection bias. It is plausible that those who entered the study share some characteristics that distinguished them from non-participants (e.g., interested in mental health or own experience with burnout). Seventh, our data collection occurred among young researchers in Flemish universities, which could affect the generalizability of our results to other countries. Nevertheless, based on former research (Coimbra Group, 2016; Levecque et al., 2017), it seems that the Flemish academic sector has significant similarities with other countries (Levecque et al., 2017). Finally, our study was conducted during the Covid-19 pandemic, which might have influenced our results, and in particular the impact of work-life interference. Nevertheless, our findings show strong similarities compared to former research carried out prior Covid-19.

## Conclusion

Our research identified five burnout profiles among young researchers: (1) High Burnout Risk (9.3%), (2) Cynical (30.1%), (3) Overextended (2.3%), (4) Low Burnout Risk (34.8%), and (5) No Burnout Risk (23.6%). Most importantly, we found a relatively high number of young researchers in the Cynical profile, which implies that young researchers are in particular vulnerable for the cynicism dimension of burnout. Additionally, work-life interference and publication pressure seemed the most significant predictors of burnout risk, while meaningfulness, social support and learning opportunities played an important protective role.

## Data Availability Statement

The raw data supporting the conclusions of this article will be made available by the authors upon request, without undue reservation.

## Ethics Statement

The studies involving human participants were reviewed and approved by the Social and Societal Ethics Committee of the KU Leuven (G-2020-2388). The patients/participants provided their written informed consent to participate in this study.

## Author Contributions

AB and LG conceptualized the design and implementation of the data collection. AB, TE, and SV wrote the manuscript. TE carried out the statistical analyses. SV and LG supervised the study. All authors contributed to the article and approved the submitted version.

## Conflict of Interest

The authors declare that the research was conducted in the absence of any commercial or financial relationships that could be construed as a potential conflict of interest.

## Publisher’s Note

All claims expressed in this article are solely those of the authors and do not necessarily represent those of their affiliated organizations, or those of the publisher, the editors and the reviewers. Any product that may be evaluated in this article, or claim that may be made by its manufacturer, is not guaranteed or endorsed by the publisher.
