# Supplementary material for: Burnout Profiles Among Young Researchers: A Latent Profile Analysis
Source: Front Psychol. 2022 May 27;13:839728. doi: 10.3389/fpsyg.2022.839728 (PMC9196046; doi:10.3389/fpsyg.2022.839728)
Supplement: Supplementary file 1 [file Table_1.DOCX]

Supplementary Material

| **Table A.1.** Means, Standard Deviations, Reliabilities (Cronbach's Alphas in Parentheses) and Pearson correlations for All Study Variables | | | | | | | | | | | | | | | | | | | | |
| --- | --- | --- | --- | --- | --- | --- | --- | --- | --- | --- | --- | --- | --- | --- | --- | --- | --- | --- | --- | --- |
| Variable | | *M* | *SD* | *1* | *2* | *3* | *4* | *5* | *6* | *7* | *8* | *9* | *10* | *11* | *12* | *13* | *14* | *15* | *16* | *17* |
| 1 | Age | 29.17 | 5.50 | - | -.08** | -.39** | .00 | -.06 | .15** | .10** | .16** | .06* | .29** | .11** | .001 | .13** | -.20** | -.03 | .10** | .09** |
| 2 | Gender (female) | 0.69 | 0.46 |  | - | .05 | .03 | -.02 | -.03 | .09** | .04 | .04 | .02 | -.06* | -.03 | .04 | .04 | .004 | .02 | .05 |
| 3 | Position (PhD) | 0.83 | 0.38 |  |  | - | .02 | .09** | -.21** | -.11** | -.08** | -.07* | -.39** | -.14** | -.03 | -.14** | .07* | .01 | -.11** | .01 |
| 4 | Emotional Exhaustion | 3.02 | 0.82 |  |  |  | (.89) | .62** | -.43** | .38** | .65** | .51** | .27** | -.32** | -.34** | -.33** | -.34** | -.35** | -.51** | .45** |
| 5 | Cynicism | 2.92 | 0.85 |  |  |  |  | (.82) | -.53** | .21** | .38** | .33** | .23** | -.29** | -.43** | -.62** | -.28** | -.38** | -.63** | .24** |
| 6 | Reduced Professional Efficacy | 3.30 | 0.56 |  |  |  |  |  | (.79) | -.13** | -.19** | -.26** | -.14** | .25** | .36** | .52** | .18** | .29** | .56** | -.14** |
| 7 | Workload | 3.22 | 0.71 |  |  |  |  |  |  | (.77) | .54** | .51** | .18** | -.26** | -.12** | -.01 | -.12** | -.24** | -.07* | .24** |
| 8 | Work-life interference | 3.01 | 0.81 |  |  |  |  |  |  |  | (.86) | .55** | .27** | -.29** | -.20** | -.12** | -.29** | -.32** | -.20** | .43** |
| 9 | Publication pressure | 3.49 | 0.71 |  |  |  |  |  |  |  |  | (.77) | .29** | -.27** | -.20** | -.14** | -.26** | -.27** | -.18** | .35** |
| 10 | Job insecurity | 2.61 | 1.11 |  |  |  |  |  |  |  |  |  | (.91) | -.11** | -.12** | -.09** | -.24** | -.17** | -.12** | .16** |
| 11 | Influence at work | 3.53 | 0.60 |  |  |  |  |  |  |  |  |  |  | (.77) | .34** | .20** | .17** | .32** | .31** | -.16** |
| 12 | Learning opportunities | 3.94 | 0.69 |  |  |  |  |  |  |  |  |  |  |  | (.82) | .45** | .22** | .37** | .42** | -.09** |
| 13 | Meaningfulness of work | 3.77 | 0.83 |  |  |  |  |  |  |  |  |  |  |  |  | (.86) | .18** | .26** | .55** | -.07* |
| 14 | Social support from colleagues | 3.76 | 0.70 |  |  |  |  |  |  |  |  |  |  |  |  |  | (.77) | .25** | .23** | -.19** |
| 15 | Social support from supervisor | 3.52 | 0.93 |  |  |  |  |  |  |  |  |  |  |  |  |  |  | (.84) | .33** | -.17** |
| 16 | Work engagement | 3.32 | 0.65 |  |  |  |  |  |  |  |  |  |  |  |  |  |  |  | (.72) | -.13** |
| 17 | Sleeping problems | 2.74 | 0.86 |  |  |  |  |  |  |  |  |  |  |  |  |  |  |  |  | (.80) |
| *Note*: Scales from 1 to 5.  * *p* < .05, ** *p* < .01. | | | | | | | | | | | | | | | | | |  |  |  |
